# Spectrum of Kidney Injury Following COVID-19 Disease: Renal Biopsy Findings in a Single Italian Pathology Service

**DOI:** 10.3390/biom12020298

**Published:** 2022-02-12

**Authors:** Alessandro Gambella, Antonella Barreca, Luigi Biancone, Dario Roccatello, Licia Peruzzi, Luca Besso, Carolina Licata, Angelo Attanasio, Mauro Papotti, Paola Cassoni

**Affiliations:** 1 Pathology Unit, Department of Medical Sciences, University of Turin, Via Santena 7, 10126 Turin, Italy; alessandro.gambella@unito.it (A.G.); angelo.attanasio@unito.it (A.A.); 2 Pathology Unit, “Città della Salute e della Scienza di Torino” University Hospital, Via Santena 7, 10126 Turin, Italy; abarreca@cittadellasalute.to.it; 3 Division of Nephrology Dialysis and Transplantation, AOU Città della Salute e della Scienza di Torino, Department of Medical Sciences, University of Turin, 10126 Turin, Italy; luigi.biancone@unito.it; 4 CMID, Coordinating Center of the Network for Rare Diseases of Piedmont and Aosta Valley, Nephrology and Dialysis Unit (ERK-Net Member), San Giovanni Bosco Hub Hospital, University of Turin, 10144 Turin, Italy; dario.roccatello@unito.it; 5 Pediatric Nephrology Unit, Regina Margherita Department, AOU Città della Salute e della Scienza di Torino, 10126 Turin, Italy; licia.peruzzi@unito.it; 6 Division of Nephrology and Dialysis, AO S. Croce e Carle di Cuneo, 12100 Cuneo, Italy; besso.l@ospedale.cuneo.it; 7 Division of Nephrology and Dialysis, ASL TO4, 10073 Ciriè, Italy; carola.licata@libero.it; 8 Pathology Unit, Department of Oncology, University of Turin, Via Santena 7, 10126 Turin, Italy; mauro.papotti@unito.it

**Keywords:** COVID-19, SARS-CoV-2, kidney biopsy, electron microscopy, minimal change disease, acute tubular necrosis

## Abstract

The onset of coronavirus disease (COVID-19) as a pandemic infection, has led to increasing insights on its pathophysiology and clinical features being revealed, such as a noticeable kidney involvement. In this study, we describe the histopathological, immunofluorescence, and ultrastructural features of biopsy-proven kidney injury observed in a series of SARS-CoV-2 positive cases in our institution from April 2020 to November 2021. We retrieved and retrospectively reviewed nine cases (two pediatric and seven adults) that experienced nephrotic syndrome (six cases), acute kidney injury (two cases), and a clinically silent microhematuria and leukocyturia. Kidney biopsies were investigated by means of light microscopy, direct immunofluorescence, and electron microscopy. The primary diagnoses were minimal change disease (four cases), acute tubular necrosis (two cases), collapsing glomerulopathy (two cases), and C3 glomerulopathy (one case). None of the cases showed viral or viral-like particles on ultrastructural analysis. Novel and specific histologic features on kidney biopsy related to SARS-CoV-2 infection have been gradually disclosed and reported, harboring relevant clinical and therapeutic implications. Recognizing and properly diagnosing renal involvement in patients experiencing COVID-19 could be challenging (due to the lack of direct proof of viral infection, e.g., viral particles) and requires a proper integration of clinical and pathological data.

## 1. Introduction

Coronavirus disease (COVID-19) is an infectious disease officially recognized as a pandemic by the WHO on 11 March 2020. It is caused by severe acute respiratory syndrome coronavirus type 2 (SARS-CoV-2), an enveloped, positive-strand RNA virus of the *Coronaviridae* family [1]. Historically, coronaviruses harbored the potential to infect humans and the peculiar ability to cause severe respiratory illness, as demonstrated by Severe Acute Respiratory Syndrome (SARS-CoV) and Middle East Respiratory Syndrome (MERS) in 2002/2003 and 2012/2013, respectively [2,3].

Since its first outbreak in Wuhan (China) in 2019, SARS-CoV-2 demonstrated a distinct airway tropism potentially leading to severe clinical manifestations (e.g., pneumonia, acute respiratory distress syndrome) and imaging confirmed lung involvement (e.g., diffuse bilateral areas of consolidation and ground–glass opacities) [4,5]. However, as the disease spread globally, reaching more than 100 million cases and 2 million deaths within a year, clinical scenarios of a broader systemic involvement affecting distinct organs emerged more clearly together with a broader spectrum of symptom severity [6,7,8,9,10,11,12,13]. To date, additional symptoms are described due to gastrointestinal (e.g., nausea, vomiting, diarrhea, and abdominal pain), hepatic (e.g., acute liver injury and cholestasis), neurological (e.g., anosmia, ageusia, encephalitis, myelitis, encephalomyelitis, stroke, and neuralgia), cardiovascular (e.g., arrhythmias, acute myocardial injury, and chronic dilated cardiomyopathy), and genitourinary (e.g., acute kidney injury, proteinuria, and hematuria) involvement [14,15,16,17,18,19,20,21,22,23,24,25]. Among these, kidneys have been significantly affected due to several SARS-CoV-2 pathophysiological elements.

SARS-CoV-2 accesses and infects human cells through its Spike protein (i.e., one of the four virion proteins) binding and interacting with the angiotensin-converting enzyme-2 (ACE-2) receptor, a molecule that is widely expressed in the respiratory, hepatic, urinary, and digestive systems. Indeed, even if the airway represents the main gateway for virus–host infection, every cell expressing the ACE-2 cell-surface receptor could be directly infected by the virus [26]. Once attached to ACE-2, the Spike protein is cleaved and the viral RNA is released within the host cell, eventually producing up to 1.000 virions per day by the infected cell [27,28,29,30]. In the kidney, the ACE-2 receptor is particularly expressed by convoluted proximal tubule epithelial cells, glomerular podocytes, and renal capillaries endothelium, where it contributes to regulating blood pressure, natriuresis, and blood volume [31,32]. In this regard, renal involvement during COVID-19 could either result from (1) a direct renal cell infection through ACE-2 receptor, (2) an indirect cytokine storm-induced injury, (3) the local tissue deposition of immune complexes of viral antigens, or (4) an endothelial damage-related injury due to microvascular dysfunction and lymphocytic endothelialitis [24,33,34]. Altogether, these conditions have made the kidney an ideal target for COVID-19-related organ injury.

Acute and chronic SARS-CoV-2 related kidney injuries are now a well-established potential development of the infection with relevant consequences both in the native and transplant setting [6,24,33,34,35,36,37,38,39,40,41,42,43,44,45,46,47,48,49,50,51,52]. Indeed, more than 40% of COVID-19 hospitalized patients have presented laboratory evidence of kidney injury (i.e., albuminuria, proteinuria, hematuria, increased creatininemia and BUN, and reduced eGFR), eventually leading to acute kidney injury (AKI) and requiring kidney replacement therapy [46,53,54].

In this setting, correctly identifying COVID-19-related morphologic features of kidney involvement will prove crucial for patient clinical management. Kidney biopsy performed during COVID-19 allows to identify a subset of morphological findings related to SARS-CoV-2 infection, such as collapsing glomerulopathy (CG) and other forms of focal segmental glomerulosclerosis, acute tubular necrosis (ATN), IgA nephropathy, thrombotic microangiopathy, crescentic glomerulonephritis, minimal change disease (MCD), membranous nephropathy, and anti-glomerular basement membrane disease (Table 1) [55,56,57,58,59,60,61,62,63,64,65,66,67,68,69,70,71,72,73,74,75,76,77,78,79,80,81,82,83,84,85,86,87,88,89,90,91,92,93,94].

Electron microscopy (EM) has also played a significant role in the COVID-19 narrative even before the pandemic, considering that the name “coronavirus” itself derived from the club-shaped spines surrounding the virus and recalling the shape of a crown (i.e., *Corona)* on EM analysis [34,95,96,97].

Based on a monocentric case series, this study aims to analyze and report the main histopathological and ultrastructural findings of SARS-CoV-2 related kidney injury to foster and improve the diagnostic awareness and the timely clinical management of these conditions.

## 2. Materials and Methods

We analyzed the medical reports and pathology files of the Città della Salute e della Scienza Hospital in Turin, from April 2020 to November 2021, to retrospectively identify cases that presented biopsy-proven kidney injury related to COVID-19. To guarantee a strict correlation with SARS-CoV-2 infection, we selected cases that (1) developed clinical signs and symptoms of kidney injury during COVID-19, (2) resulted in a positive to SARS-CoV-2 test on admission and (3) were positive at the time of the biopsy. We retrieved 9 cases that satisfied these criteria and then collected the corresponding clinical and pathology data to create a database anonymized by a pathology staff member not involved in the study before starting any analysis.

Original slides and ultrastructural images were retrieved and reviewed by expert renal pathologists. At the time of diagnosis, histopathological, immunohistochemical (IHC), and immunofluorescence (IF) analyses were performed according to our laboratory clinical protocols, as previously described [98]. IHC and IF antibodies are detailed in Supplementary Methods. EM was performed by adopting standard protocols, thus requiring glutaraldehyde-based fixation and epoxy resin embedding. Then, single ultrathin sections 50–100 nm thick were obtained and analyzed using the Philips CM10 transmission electron microscope (Philips Electronics, Eindhoven, The Netherlands).

## 3. Results

### 3.1. Overall Population

Our series was composed of nine cases developing kidney injury during SARS-CoV-2 infection. The most represented kidney-related clinical manifestation was nephrotic syndrome (five cases) followed by AKI (two cases). One case presented combined AKI and nephrotic syndrome, and one case was a mild clinical silent renal involvement characterized by microhematuria and leukocyturia. Males and females were almost equally represented (five and four cases, respectively) and the median age was 64 (interval: 15–80). Most cases were Caucasian (seven), whereas two cases were Afro-American.

Regarding the histopathological analysis, most cases presented features of minimal change disease (MCD) (four), followed by acute tubular necrosis (ATN) (two) and collapsing glomerulopathy (CG) (two). One case only presented C3 glomerulonephritis (C3G). Cases 2 and 4 presented overlapping features (ATN/microangiopathic glomerular injury and ATN/CG, respectively) and they were classified according to the prevalent pattern observed (ATN and CG). Light microscopy (LM) and immunofluorescence (IF) were always performed, whereas electron microscopy (EM) was not performed in one case due to tissue exhaustion. Case 1 required immunohistochemical stains (IHC) for C4d and SV40 to exclude post-transplant complications, whereas C4d was also performed in Case 9 for diagnostic differential purposes. Additionally, Caveolin-1 (Cav1) was tested for in Case 2 as a marker of thrombotic microangiopathy (TMA).

Clinical and pathology data are summarized in Table 2.

### 3.2. Acute Tubular Necrosis (ATN)

Cases developing ATN were a male (Case 1) and a female (Case 2) in their 70s.

**Figure 1:**
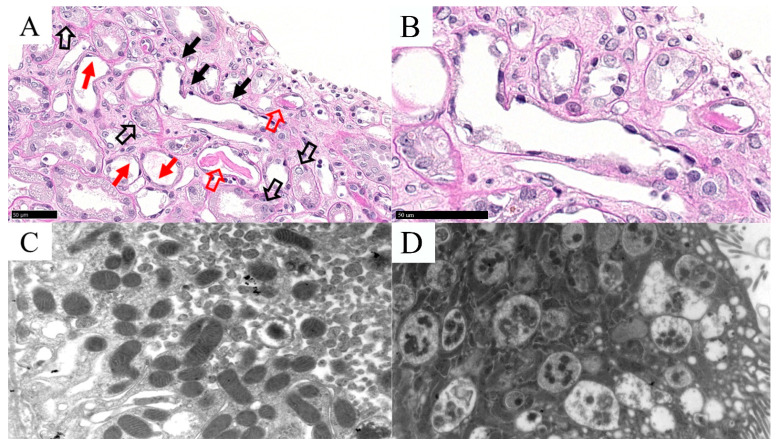
Histomorphological and ultrastructural features of Case 1. PAS stain (**A**) highlighted epithelial cell death (i.e., loss of or pale staining nuclei and eosinophilic cytoplasm; full black arrow), nuclei regenerative atypia (empty black arrow), and reduced or absent brush borders (full red arrow); eosinophilic casts in tubular lumens were also observed (empty red arrow) containing nuclear and cytoplasmic debris originated from necrotic tubular epithelial cells. Details of an injured tubules of the same area at high-power magnification (**B**). EM analysis confirmed the acute tubular injury showing mitochondrial condensation (**C**), and increased phagolysosomes (**D**).

Case 1 had no features of glomerular injury apart from a focal and minimal ischemia-related glomerulosclerosis, whereas the tubular compartment resulted in being seriously injured, presenting thinning and loss of apical cytoplasm and reactive nuclear atypia (Figure 1).

IHC stains for C4d and SV40 were negative as well as IF analyses (i.e., immunoglobulin heavy-chains, complement fractions, and fibrinogen). Additionally, EM analysis was performed to exclude ultrastructural signs of chronic rejection (the patient underwent kidney transplantation two years before) and further confirmed the observed histopathological findings. EM had the characteristic features of ATN, such as tubular membrane apical blebs and shedding, mitochondrial condensation, and increased phagolysosomes (Figure 1). No viral particles were observed on EM, as well as no signs of chronic rejection (donor specific antibody (DSA) tested negative since the day of transplantation). Based on this evidence and according to the clinical history, a COVID-19-related ATN diagnosis was performed.

**Figure 2:**
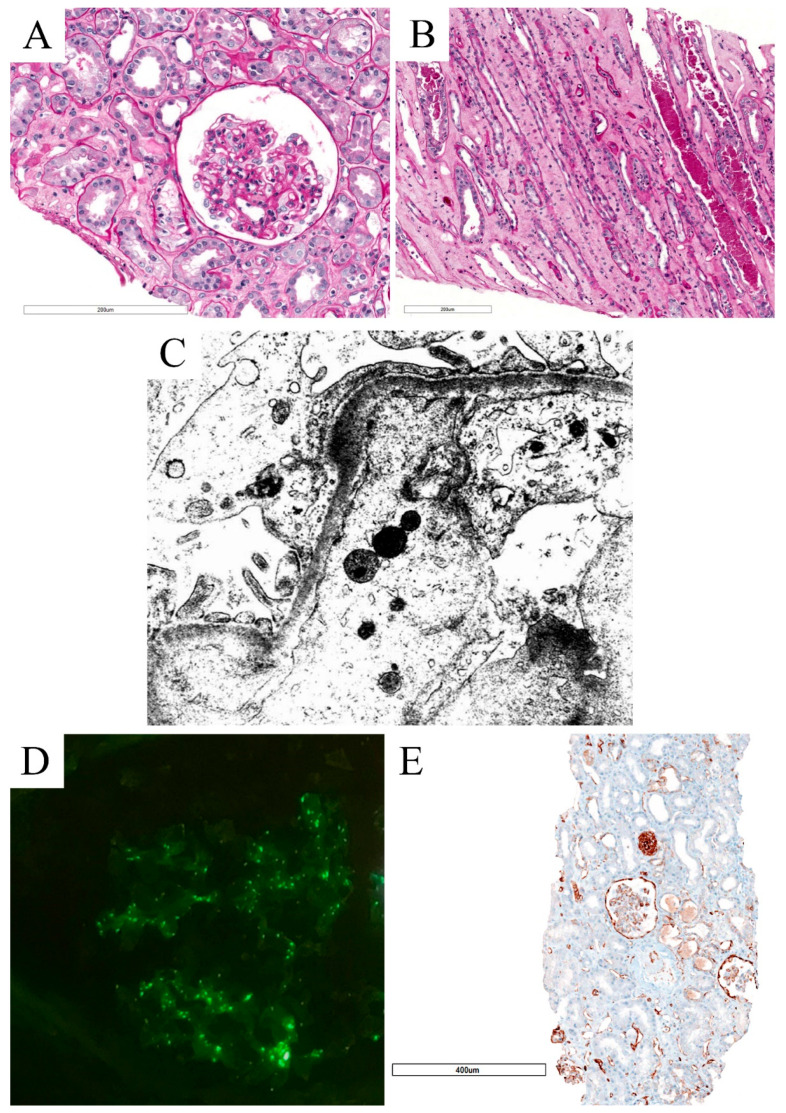
Histomorphological, IF, and ultrastructural features of Case 2. PAS stain (**A**,**B**) showing segmental wrinkling of glomerular basement membranes (**A**) and details of ATN (flattened epithelial cells and denudation of tubular basement membrane with necrotic material in lumen; (**B**). EM analysis showed segmental foot process effacement and confirmed the collapsed glomerular basement membrane with mild subendothelial space widening and loss of endothelial cell fenestrae (**C**), whereas IF showed a mild and unspecific positivity for C3 (**D**). Cav1 immunohistochemistry showed diffuse positivity both in peritubular capillaries and glomeruli (**E**).

Case 2 also presented tubular features suggestive of ATN but with associated glomerular injury. Indeed, glomeruli showed a focally collapsed basement membrane, suggesting a mild glomerular microangiopathic injury (Figure 2).

IF showed a mild scattered finely granular positivity for C3 complement fragment that was considered unspecific (Figure 2). EM analysis identified segmental foot process effacement, and wrinkling of glomerular basement membrane with mild subendothelial electron-lucent widening and loss of endothelial cell fenestrae, thus confirming features of mild TMA glomerular injury (Figure 2). No electron-dense deposits and viral particles were observed on EM. Caveolin-1 (Cav1) immunohistochemistry demonstrated diffuse positivity both in peritubular and glomerular capillaries, as previously reported by our group in TMA [98].

### 3.3. Collapsing Glomerulopathy (CG)

Two cases (Case 3 and Case 4) presented histopathological features of CG that affected two middle-aged Afro-American patients (61 and 45 years old, respectively).

**Figure 3:**
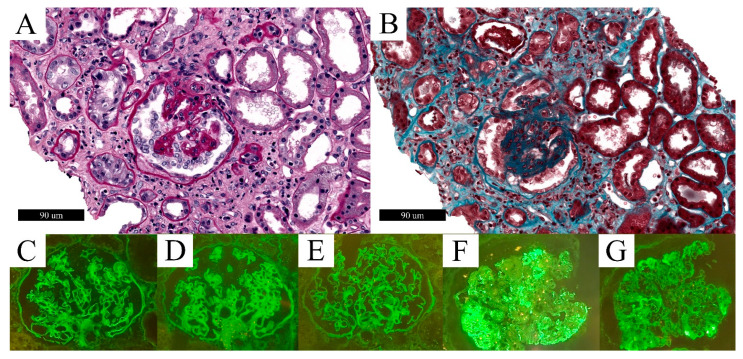
Histomorphological and IF features of Case 3. PAS stain (**A**) showed global collapse of glomerular tuft with overlying hypertrophy and hyperplasia of podocytes, highlighted by trichrome stain (**B**). IF showed mild and diffuse linear positivity for IgG (**C**), Lambda light chain (**D**), and Kappa light chain (**E**), while IgM (**F**) and C3 (**G**) were positive in sclerotic areas.

A kidney biopsy of Case 3 showed features of glomerular injury, such as global or segmental glomerular tuft collapse with several hypertrophic podocytes, rarely containing eosinophilic protein droplets (Figure 3).

EM confirmed a glomerular segmental sclerosis (involving approximately 20% of capillary loops) characterized by the collapse of glomerular capillaries and prominent podocyte hypertrophy, and containing electron-dense protein resorption droplets and massive foot process effacement. There was also a diffuse thickening of glomerular basement membranes (mean thickness: 950 nm) and a moderate mesangial matrix deposition. No electron-dense deposits nor viral particles were observed on EM. Overall, these features were consistent with a diagnosis of focal segmental glomerulosclerosis, collapsing variant, namely CG, with associated diabetic glomerulosclerosis (grade IIb according to *Tervaert* et al. [99]), confirmed by IF analysis (Figure 3). Indeed, IgG and Kappa and Lambda light chains showed a mild diffuse linear positivity of glomerular and tubular basement membrane on IF, whereas focal and segmental positivity for IgM, C3, and C1q was observed in sclerotic areas (Figure 3).

The kidney biopsy of Case 4 presented similar features, and was characterized by global or segmental glomerular capillary loop collapse with prominent podocyte hyperplasia and hypertrophy. Additionally, there was also a moderate acute tubular injury. IF stained slides could not be evaluated because only one single glomerulus with global sclerosis was present, whereas EM analysis could not be performed due to tissue exhaustion. Based on LM features and clinical data, a diagnosis of CG with associated moderate ATN features was rendered.

### 3.4. Minimal Change Disease (MCD)

Four cases presented MCD on kidney biopsy, none of them with significant or specific LM findings (Table 3).

**Figure 4:**
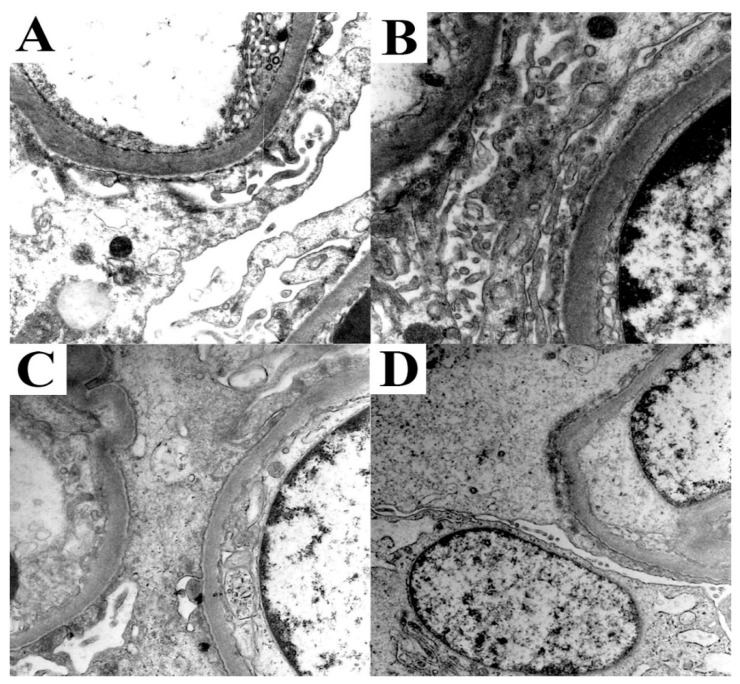
Ultrastructural features of MCD. Case 5 (**A**), Case 6 (**B**), Case 7 (**C**), and Case 8 (**D**) presented features of diffuse FPE.

Two cases presented mild age-related glomerulosclerosis and rare signs of ATN in the regenerative phase (Case 6), and minimal glomerular basement membrane wrinkling with occasional regenerative features of ATN (Case 7). Accordingly, no specific IF pattern was observed in any case. On EM analysis, all cases showed diffuse FPE involving up to 80% of glomerular capillary loops (Table 3) (Figure 4).

### 3.5. C3 Glomerulonephritis (C3G)

**Figure 5:**
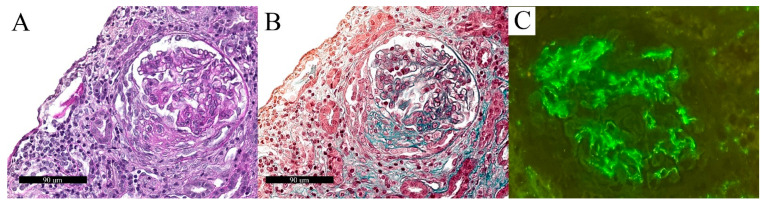
Histomorphological and IF features of Case 9. PAS stain (**A**) showed a glomerulus with fibrocellular crescent, highlighted by trichrome stain (**B**). IF for C3 was positive, showing a diffuse and strong granular positivity in glomerular basement membranes and mesangium (**C**).

The last case (Case 9) referred to a pediatric patient that presented C3G. In this case, fibroepithelial or cellular crescents were detected in >50% of glomeruli (Figure 5).

Crescents were either circumferential, leading to global glomeruli collapse, or partial, inducing segmental collapsed glomeruli basement membranes (Figure 5). We also observed focal features of ATN in the presence of erythrocyte casts. IF demonstrated diffusely and strongly granular positivity for C3 in glomerular basement membranes and mesangium (Figure 5). C4d IHC stain resulted negative, further confirming the activation of the alternative complement pathway. EM analysis showed amorphous deposits, mainly localized in mesangial and paramesangial areas and characterized by intermediate density and sometimes indistinct borders (Figure 5). No viral particles were observed on EM. Based on these findings and according to the clinical background, the diagnosis of C3G was established.

## 4. Discussion

This study described the pathology findings of COVID-19-associated kidney injury reported in our institution since the pandemic outbreak, thus depicting the main morphologic, immunofluorescence, and ultrastructure features we reported in our daily diagnostic routine. In particular, we (1) confirmed the kidney involvement during SARS-CoV-2 infection, affecting all ages and both native and transplanted organs regardless of lung symptoms, (2) highlighted the relevance of clinical correlation rather than the presence of viral particles/molecular evidence to diagnose COVID-19-related kidney injury, (3) described patterns of kidney injury as previously observed (i.e., ATN and CG), and (4) reported additional forms of kidney involvement during COVID-19 (i.e., MCD and C3G).

Clinical correlation is crucial to define and properly characterize kidney involvement during COVID-19. At first, COVID-19 emerged as an acute condition targeting the lungs and mainly affecting adult patients. To date, we know that the disease is more multifaceted, presenting a wide range of symptom severity and affecting all ages. In addition, several organs are involved but the kidney has emerged as a peculiar site of injury. Indeed, in a prospective cohort study enrolling approximatively 700 patients, Cheng et al. observed that more than 40% of COVID-19 hospitalized patients presented evidence of kidney injury and further reported that, similarly to H1N1 influenza A virus and SARS-CoV, AKI resulted in an independent risk factor for hospital mortality [46]. Similarly, Chan et al. reported that 46% of 3993 COVID-19 hospitalized patients in the US developed AKI, whereas 19% required kidney replacement therapy [53]. Data published by Karagiannidis et al. from a multicentric German cohort of 10,021 COVID-19 hospitalized patients reported a renal failure rate of 23% and dialysis percentage ranging from 6% (all patients) to 27% (within the ventilated patient subgroup) [54].

Further addressing the renal involvement during COVID-19, two considerations are noteworthy. Firstly, COVID-19 could manifest with extrapulmonary symptoms only and, in particular, with signs of kidney involvement in an otherwise clinically silent setting [63]. Although our study focused on histomorphological features, most cases did not present lung-related symptoms or an abrupt manifestation during the disease. This finding is particularly relevant to properly define the role of SARS-CoV-2 in determining kidney injury and eventually prevent delayed diagnosis. The tropism of SARS-CoV-2 for renal cells was initially assumed based on the well-documented elevated expression of ACE-2 receptor in the kidney, but recent findings suggested additional (and probably more relevant) mechanisms of damage, such as cytokine storm, immune complexes deposition, and (micro) vascular dysfunction [24,33,34]. Indeed, although some case reports and autopsy findings reported putative SARS-CoV-2 particles on EM kidney analysis [72,100], further studies addressing both ultrastructural and molecular evidence of the virus excluded its direct presence in kidney sites of injury [58,76,96,101,102,103,104,105]. Accordingly, we did not observe any viral or viral-like particles on EM analysis, thus supporting the emerging concept that the direct demonstration of viral presence, either ultrastructural or molecular, should not be considered mandatory to diagnose a COVID-19-related kidney injury, whereas temporal and clinical correlation proved to be more relevant.

This is significant evidence, as it could refine the concept of COVID-19-related kidney involvement, thus identifying all the morphological patterns associated with SARS-CoV-2 infection and ultimately improving the diagnosis and clinical management of affected patients. Secondly, we confirmed the renal impairment in the pediatric population. Pediatric manifestations of SARS-CoV-2-mediated kidney injury are poorly described. Based on published data, the clinical scenario did not differ substantially from the adult setting, thus presenting high rates (up to 37%) of AKI development, thrombotic microangiopathy, and acute necrotizing glomerulopathy [106,107,108,109,110,111,112]. Our series, although limited, included two pediatric patients that presented unusual patterns of SARS-CoV-2 pediatric injury, namely MCD and C3G. In the pediatric population, MCD is primarily associated with nephrotic syndrome [113] and is characterized by diffuse FPE on EM analysis, combined with the almost complete absence of LM findings and the unspecific focal IF expression of IgM, as we observed in our pediatric MCD case. MCD etiopathogenesis is mainly idiopathic, but it could be secondary to different pathologies, including viral infection, as reported for COVID-19 hospitalized adult patients [56,71,80,114]. Indeed, MCD could develop following a “two-hit” mechanism characterized by podocytes increased and unregulated CD80 expression that is triggered by viral products [115,116,117].

Similarly, C3G could also be related to viral infection. C3G is part of the primary membranoproliferative glomerulonephritis, a group of pathologies recently re-classified to properly represent the etiopathogenesis and improve the subsequent treatment accordingly [118]. C3G could manifest as a nephritic or nephrotic syndrome, but the most common clinical presentation is asymptomatic hematuria or proteinuria (60% of cases, approximatively) [119,120], as observed in our case. Pathognomonic features of this condition were represented by LM-evident glomerular deposition further characterized as an intense C3-positive IF pattern associated with electron-dense deposits on EM. Accordingly, these features characterized the C3G COVID-19-related case reported in this study.

These cases are of particular interest, as they expand our current knowledge of COVID-19-related pediatric kidney diseases. Overall, COVID-19 pediatric cases showed excellent outcomes compared to adults as they are characterized by reduced incidence of acute organ failure (less than 1% of hospitalized patients, approximatively) [111,121,122,123,124,125,126,127]. However, children experiencing COVID-19 presented a clinically subtle kidney involvement that was challenging to recognize and diagnose, particularly in extrapulmonary-only clinical settings [111]. Importantly, misdiagnosing kidney injury in the pediatric population is likely to lead to delayed treatment and increased development of long-term complications. In the adult setting, COVID-19-related kidney injury started being increasingly reported as a heterogeneous and evolving scenario, but two histomorphological features emerged as particularly relevant, namely ATN and CG. ATN is referred to as reversible destruction of the excretory compartment of the kidney, the tubules. Several conditions could lead to ATN, but two significant groups were usually described: (1) ischemia-related, as observed during marked hypotension/shock conditions, such as acute pancreatitis and trauma, and (2) nephrotoxic-related agents (e.g., drugs (gentamycin, cisplatin, methotrexate), hemoglobin, myoglobin). As per the COVID-19 infection, ATN nowadays represents a well-described complication of COVID-19 hospitalized patients and the most common histomorphological pattern leading to AKI [101,128,129,130,131,132,133,134,135]. In our series, we observed a relevant rate of ATN in different phases (from active necrosis to later regenerative stages), and, according to the literature, it was the main pattern related to AKI. ATN diagnosis is mainly LM-based and characterized by a severe involvement of the tubule and a relative sparing of the glomerular compartment, as observed in our series. Of note, one of our ATN cases was observed in a kidney-transplant patient. COVID-19 effects on transplant patients are particularly relevant, as these patients are treated with chronic immunosuppression protocols and usually harbored individual predisposing factors for kidney injury [136]. In particular, COVID-19 kidney-transplant recipients showed worse outcomes than the general populations, characterized by a more aggressive clinical course and an increased mortality rate [38,64,104,136,137,138]. From a pathology perspective, the biopsy findings consistently showed features of rejection, particularly acute rejection (both cellular and antibody-mediated), or transplant-related condition, such as calcineurin toxicity [64,136,138]. In contrast, we did not report any morphological and ultrastructural sign of rejection, but rather features of tubular injury and necrosis, further supporting the aggressive course in the transplant subgroup of patients.

CG is an additional and relevant emerging condition associated with COVID-19 disease with crucial clinical implications [63]. Indeed, CG represented a rapidly degenerative subset of focal segmental glomerulosclerosis leading to nephrotic syndrome, organ dysfunction, and renal failure. CG was particularly related to the Afro-American ancestry due to a characteristic apolipoprotein L1 gene polymorphism and is mainly caused by a viral-related inflammatory cascade [139]. Of note, CG etiopathogenesis, pathology features, and the rise of incidence observed during the COVID-19 pandemic has led to the definition of COVID-19-associated nephropathy (COVAN) due to the resemblance with the features observed during the HIV pandemic [63,72,76,140]. The main histomorphological findings of CG are glomerular collapse (either global or segmental) together with podocyte hyperplasia and hypertrophy, with IgM and C3 in sclerotic areas on IF analysis. EM proved helpful in confirming glomerular involvement as capillary loop collapse and diffuse FPE. In our series, we confirmed both the pathology findings and demographic trend, considering that the currently reported cases of CG occurred in Afro-American patients.

To address our study limitations, mainly represented by the small sample size and the limited insights on injury mechanistic, further studies should explore multicentric larger series providing more granular molecular analysis and identifying the specific pathways activated during COVID-19-related kidney injury. 

## 5. Conclusions

COVID-19 is a constantly evolving disease, and new variants of SARS-CoV-2 are being reported. Kidney manifestations of COVID-19 should be carefully and specifically diagnosed to guide patient clinical management and prevent chronic organ injury, particularly considering the fragile subgroups of patients (e.g., pediatric and transplanted populations) that could be affected and the relatively poor direct signs of viral infection (i.e., viral particles/molecular footprint) in tissue samples.

## Figures and Tables

**Table 1:** Pathology features of kidney involvement in COVID-19.

Pathology Features	Reference
Collapsing glomerulopathy (now defined as COVID-19–associated nephropathy (COVAN))	[56,57,58,61,62,64,66,69,70,71,72,73,74,75,76,77,78,79,80,81,82]
Acute tubular necrosis	[58,64,66,69,71,72,76,80,82,83,84]
IgA nephropathy	[66,69,81,85,86,87]
Thrombotic microangiopathy	[58,64,66,67,69]
Crescentic glomerulonephritis	[58,66,69,88,89]
Non collapsing FSGS	[64,66,69,84]
Minimal change disease	[64,69,71,80]
Membranous nephropathy	[66,69,71,81]
Anti-glomerular basement membrane disease	[69,71,90,91]
Diabetic nephropathy	[64,69,81]
Myoglobin cast nephropathy	[58,69]
Post-infectious glomerulonephritis	[64,69]
Antibody-mediated rejection	[64,69]
Light chain cast nephropathy	[66,69]
Calcineurin inhibitor nephrotoxicity	[66]
Amyloidosis	[66,69]
Cortical infarction	[69,71]
Lupus nephritis	[69,71]
T-cell mediated rejection	[69,71]
Acute interstitial nephritis	[69,91]
Oxalate nephropathy	[92]
Granulomatous tubulointerstitial nephritis	[93]
Membranoproliferative glomerulonephritis	[94]

**Table 2:** Clinical and pathology data of our series.

Cases	Sex/Age	Risk Factor/Clinical History	Clinical Background on Admission *	Kidney Impairment	Laboratory Data ^§^	Analysis Performed	Pathology Features
Case 1	M/73	Kidney transplant; hypertension; metabolic syndrome	Elevated fever	AKI	Creatininemia: 4.0 mg/dL Proteinuria: 0.64–1 gr/die	LM; IHC; IF; EM	ATN
Case 2	F/77	Hypertension; obesity; rheumatoid arthritis	Pneumonia	AKI	Creatininemia: 3.2 mg/dL Proteinuria: 0.17 gr/die	LM; IF; EM	ATN and TMA
Case 3	F/61	Hypertension; obesity; type 2 diabetes	Acute respiratory distress syndrome	Nephrotic syndrome	Proteinuria: 14 gr/die Albuminemia: 2.2 g/dL	LM; IF; EM	CG
Case 4	M/45	Hypertension; type 2 diabetes	Fatigue; dyspnea	Nephrotic syndrome	Proteinuria: 30 gr/die	LM; IF	ATN and CG
Case 5	M/15	None	Fever; fatigue; peripheral edema	Nephrotic syndrome	Proteinuria: 1.83 gr/die/1.73 m^2^ Albuminemia: 3.9 g/dL	LM; IF; EM	MCD
Case 6	M/71	Hypertension; obesity;	Pneumonia; peripheral edema	Nephrotic syndrome	Proteinuria: 15.3 gr/die Albuminemia: 2.3 g/dL	LM; IF; EM	MCD and ATN
Case 7	F/80	Hypertension	Peripheral edema	Nephrotic syndrome	Proteinuria: 3.8 gr/die Albuminemia: 2.5 g/dL	LM; IF; EM	MCD and ATN
Case 8	M/64	Hypertension	Peripheral edema	AKI and Nephrotic syndrome	Creatininemia: 2.8 mg/dL Proteinuria: 22 gr/die Albuminemia: 2.7 g/dL	LM; IF; EM	MCD
Case 9	F/15	None	Fever	Microhematuria; leukocyturia	Creatininemia: 1.1 mg/dL eGFR 60 mL/min/1.73 m^2^ Proteinuria: 1.9 gr/die/1.73 m^2^ Albuminemia: 3.7 g/dL ANCA negative	LM; IHC; IF; EM	C3G and ATN

*: All patients were tested for SARS-CoV-2 and resulted in being positive on admission and at the time of the biopsy; ^§^: at the time of the biopsy. LM: light microscopy; IHC: immunohistochemistry; IF: immunofluorescence; EM: electron microscopy; ATN: acute tubular necrosis; TMA: thrombotic microangiopathy; CG: collapsing glomerulopathy; MCD: minimal change disease; C3G: C3 glomerulonephritis.

**Table 3:** Histomorphological and ultrastructural features of MCD.

Cases	Histological Findings	IF Pattern	EM Findings— Percentage of Foot Process Effacement (FPE)
Case 5	No evident injury	Unspecific faint mesangial IgM positivity	80%
Case 6	Focal mild ischemic global glomerulosclerosis age-correlated and occasional nuclear tubular atypia	Negative	77%
Case 7	Minimal and focal glomerular basement membrane wrinkling and occasional nuclear tubular atypia	Negative	75%
Case 8	No evident injury	Negative	70–75%

## Data Availability

The data presented in this study are available on request from the corresponding author. The data are not publicly available due to privacy restriction.

## Supplementary Materials

The following are available online at https://www.mdpi.com/article/10.3390/biom12020298/s1. Supplementary Methods: IHC and IF protocols and procedures.
