# Supplementary material for: Spectrum of Kidney Injury Following COVID-19 Disease: Renal Biopsy Findings in a Single Italian Pathology Service
Source: Biomolecules. 2022 Feb 12;12(2):298. doi: 10.3390/biom12020298 (PMC8961620; doi:10.3390/biom12020298)
Supplement: Supplementary file 1 [file biomolecules-12-00298-s001.zip › biomolecules-1574509-supplementary.pdf]

Supplementary Methods. Immunohistochemical (IHC) stains were performed with the automated immunostainer BenchMark XT AutoStainer® (Ventana Medical Systems, Arizona, USA) using antibodies against C4d (clone SP91; Ventana Medical Systems), SV40 (clone MRQ-4; Cell Marque), and Caveolin-1 (clone N-20; Santa Cruz Biotechnology, Santa Cruz, CA).

Direct immunofluorescence (IF) was performed on three μm-thick fresh-frozen tissue cut slides, fixed for 10 minutes in cold acetone, and then stained with a panel of FITC-conjugated antibodies (IgA, IgM, IgG, C3, C4, C1q, and fibrinogen; New Scientific Company, Lombardy, Italy).

All the procedures mentioned in the manuscript were performed according to manufacturer's protocols and instructions.
